# Effect of a Protein Supplement on the Gut Microbiota of Endurance Athletes: A Randomized, Controlled, Double-Blind Pilot Study

**DOI:** 10.3390/nu10030337

**Published:** 2018-03-10

**Authors:** Diego Moreno-Pérez, Carlo Bressa, María Bailén, Safa Hamed-Bousdar, Fernando Naclerio, Manuel Carmona, Margarita Pérez, Rocío González-Soltero, Maria Gregoria Montalvo-Lominchar, Claudia Carabaña, Mar Larrosa

**Affiliations:** 1 Departamento de Educación, Métodos de Investigación y Evaluación, Universidad Pontificia de Comillas, ICAI-ICADE, Cantoblanco, Madrid 28015, Spain; diego.moreno@allinyourmind.es; 2 Facultad de Ciencias Biomédicas, Universidad Europea de Madrid, Villaviciosa de Odón, Madrid 28670, Spain; carlo.bressa@universidadeuropea.es (C.B.); maria.bailen@universidadeuropea.es (M.B.); mariadelrocio.gonzalez@universidadeuropea.es (R.G.-S.); 3 Escuela de Doctorado e Investigación, Universidad Europea de Madrid, Villaviciosa de Odón, Madrid 28670, Spain; safahamed1994@gmail.com (S.H.-B.); manuel.carmona@universidadeuropea.es (M.C.); margarita.perez@universidadeuropea.es (M.P.); mariagregoria.montalvo@universidadeuropea.es (M.G.M.-L.); claudiacarabana@hotmail.com (C.C.); 4 Department of Life and Sports Sciences, University of Greenwich, Kent ME4 4TB, UK; f.j.naclerio@greenwich.ac.uk

**Keywords:** sport supplements, fecal ammonia, *Bifidobacterium longum*, fecal pH, branched short-chain fatty acids

## Abstract

Nutritional supplements are popular among athletes to improve performance and physical recovery. Protein supplements fulfill this function by improving performance and increasing muscle mass; however, their effect on other organs or systems is less well known. Diet alterations can induce gut microbiota imbalance, with beneficial or deleterious consequences for the host. To test this, we performed a randomized pilot study in cross-country runners whose diets were complemented with a protein supplement (whey isolate and beef hydrolysate) (*n* = 12) or maltodextrin (control) (*n* = 12) for 10 weeks. Microbiota, water content, pH, ammonia, and short-chain fatty acids (SCFAs) were analyzed in fecal samples, whereas malondialdehyde levels (oxidative stress marker) were determined in plasma and urine. Fecal pH, water content, ammonia, and SCFA concentrations did not change, indicating that protein supplementation did not increase the presence of these fermentation-derived metabolites. Similarly, it had no impact on plasma or urine malondialdehyde levels; however, it increased the abundance of the *Bacteroidetes* phylum and decreased the presence of health-related taxa including *Roseburia*, *Blautia*, and *Bifidobacterium longum*. Thus, long-term protein supplementation may have a negative impact on gut microbiota. Further research is needed to establish the impact of protein supplements on gut microbiota.

## 1. Introduction

The World Health Organization recommendation for protein intake in adults, considering a net nitrogen balance, is 0.83 g/kg body weight/day. Because athletes have a greater nutrient expenditure, the American Dietetic Association, the Dietitians of Canada, and the American College of Sports Medicine recommend an intake range from 1.2 to 2 g/kg body weight/day for endurance- and strength-trained athletes [1]. These recommendations are not only based on the net nitrogen balance, they also seek to promote muscle mass and improve performance [2,3]. Several studies have shown that intake of protein supplements increases muscle mass and improves sports performance [4,5], providing context for their popularity [6]; however, their effects on organs other than muscle have been poorly studied. A factor that can be modified by the increase in dietary protein intake is the gut microbiota, which may have positive or negative repercussions on host health [7].

The gut microbiota is an important factor that shapes both energy harvest and storage through the metabolism of proteins and the production of several metabolites: branched short-chain fatty acids (SCFAs), ammonia, sulfur-containing metabolites such as hydrogen sulfide and methanethiol, and neuroactive compounds such as tryptamine, serotonin, phenethylamine, and histamine [8,9]. Some of these metabolites are related to deleterious effects on health; for instance, ammonia and hydrogen sulfide favor colon cancer and inflammatory bowel disease [10,11], whereas others such as branched SCFAs have hitherto unknown effects [12]. Moreover, the gut microbiota can also synthesize de novo amino acids, and thus the balance between production and digestion determines the level of nitrogen in the body [13]. Animal studies have revealed bidirectional communication between the gut microbiota and the muscle, in which gut microbiota can affect muscle energy homeostasis by interfering with fat deposition and lipid and glucose metabolism through various metabolites including SCFAs and secondary bile salts [14]. Likewise, physical activity may induce changes in gut microbiota through several factors: myokines released during exercise or changes to intestinal transit generated by exercise [14,15] may promote the growth of certain species beneficial to health [15,16].

Supplementation with powder-hydrolyzed beef protein in resistance-training males can significantly increase the biceps brachialis thickness and arm circumference compared with carbohydrate or whey protein supplementation [17]. In addition, a decline in fat mass and an increase in fat-free mass can be observed after eight weeks of supplementation with 135 g of lean beef in healthy subjects participating in a resistance-training program [18]. The effect of protein supplementation on the microbiota of endurance athletes has, however, not yet been studied. The aim of the present study was to determine the changes produced by a high-quality hydrolyzed beef and whey protein supplement on gut microbiota, fecal pH, and metabolites derived by protein consumption such as SCFAs and ammonia.

## 2. Materials and Methods

### 2.1. Study Design

The study was in the context of a pilot randomized controlled parallel intervention designed to fulfill the Consolidated Standards of Reporting Trials statement (CONSORT) criteria. The study protocol was in accordance with the Ethics Guidelines of the Declaration of Helsinki and was approved by the Ethics Committee for Clinical Research of the Comunidad de Madrid (Spain), code 2016 RM/05. The study is registered in Clinicaltrials.gov with the accession number NCT02425020. It was conducted between September 2016 and January 2017. Pre-evaluations were carried out during September, followed by 10 weeks of supplementation, and post-evaluations were performed in December 2016.

### 2.2. Subjects and Dietary Supplementation

A total of 24 participants were recruited according to the following inclusion criteria: regularly endurance training male, aged from 18 to 45 years, with a minimum of five years of regular endurance training and a frequency of five sessions per week minimum, and a minimum total weekly training time of 240 min. Because some studies have reported gender-associated differences in gut microbiota and in diet response [19,20], male gender was selected as an inclusion criterion to decrease data variability in the pilot study. Exclusion criteria were any musculoskeletal injury, metabolic conditions, any diseases, chronic use of medications, smoking, or use of nutritional supplements (e.g., iron, creatine, whey protein, amino acids, or derivative compounds such as L-carnitine) within eight weeks prior to the start of the study. All participants signed a written informed consent form. After completing the first assessment session, each participant was given a batch of one of the two products, assigned according to randomization: the control (CHO) group received maltodextrin (*n* = 12), which is absorbed in the upper part of the intestine and does not reach the colon; the protein (PRO) group received a blend of whey isolate (10 g) and beef hydrolysate (10 g) (*n* = 12). The two supplements were presented as 24-g sachets of powder for each intake, which had to be diluted in ~200 mL of commercial orange drink to mask the supplement’s flavor. The commercial drink only contained 5% orange, and thus the phytochemical content was negligible. The mixed drinks were similar in appearance, texture, and taste, and were isoenergetic. The administered amount of protein is within the 90% confidence interval (180–300 mg·kg^−1^), beyond which there is no further increase in muscle protein synthesis in young men [21] during relative resting conditions [22,23]; this quantity has been shown to be effective after exercise [24] irrespective of training status [25]. Supplements were consumed once per day after training during the training days or before breakfast during non-training days, for 10 weeks. The nutritional composition of each product is shown in Table 1.

Tolerance, collected from any adverse events, and compliance with supplement intake (determined by individual follow-up) were evaluated continuously during the intervention. Only those participants who completed the 70 days of supplementation intake with a minimum training frequency of four sessions per week (40 workouts in total) were included in the analysis. Participants verbally confirmed that they maintained their habitual diet throughout the trial period.

Fecal and urine samples and food frequency questionnaires (to assess that habitual diet was maintained) were taken initially (*t* = 0) and after the 10 weeks of supplementation (*t* = 10 weeks). First morning urine and fasting blood samples were collected, centrifuged at 3000× *g* for 10 min, and stored at −80 °C until use.

### 2.3. Anthropometry and Body Composition

Height and weight were measured with a tallimeter (Asimed T2, Barcelona, Spain) and a balance scale (Ano Sayol SL, Barcelona, Spain), respectively; body mass index (BMI) was calculated as weight (kg)/height (m^2^). Body composition was evaluated by dual-energy X-ray absorptiometry (Lunar iDXA, GE Healthcare, Madison, WI, USA). The measures of body composition were as follows: total body fat mass, estimated visceral adipose tissue (VAT), total muscle mass, and fat and muscle mass distribution in the trunk and extremities. The following indices were calculated using the obtained values: adiposity index (AI) = total fat/height^2^; muscular mass index (MMI) = total muscle mass/height^2^ and appendicular muscular mass index (AppMMI) = muscle mass in arms + legs/height^2^, fat free mass index (FFMI) = BMI − fat mass.

### 2.4. Food Frequency Questionnaire

Participants’ dietary pattern characterization was carried out using a food frequency questionnaire (FFQ) with 93 food items. The FFQ was given to participants at the beginning and at the end of the intervention period. Data from the FFQ were analyzed using Dietsource software 3.0 (Novartis, Barcelona, Spain) to obtain the total ingested amount of carbohydrates, protein, fats, and fiber, and the total energy ingested.

### 2.5. Stool Collection and Bacterial DNA Extraction

Participants were provided with a Fe-Col^®^ Fecal Sample Collection Kit (Alpha Laboratories, Eastleigh UK) plus an icebox and a cooler to maintain the samples at −20 °C until delivery to the laboratory, where samples were aliquoted and stored at −80 °C. Bacterial DNA was extracted from 100 mg of sample using the commercial E.Z.N.A.^®^ Stool DNA Kit (Omega Biotek, Norcross, GA, USA) and a bead-beating homogenizer (Bullet Blender Storm, Next Advance, Troy, NY, USA). The concentration and purity of DNA were measured using a Quant-iT PicoGreen dsDNA Assay Kit (ThermoFisher Scientific, Waltham, MA, USA) and an FP-8300 spectrofluorimeter (Jasco, Tokyo, Japan).

### 2.6. Sequencing and Bioinformatics

For sequencing, a DNA fragment comprising the bacterial hypervariable regions V3 and V4 of 16S rRNA gene was amplified using the primer pair 5′-TCGTCGGCAGCGTCAGATGTGTATAAGAGACAG-3′ and 5′-GTCTCGTGGGCTCGGAGATGTGTATAAGAGACAG-3′ [26]. The amplicon of 459 bp was visualized in a 0.8% agarose gel stained with ethidium bromide, and bands were cut and cleaned using the MinElute Gel Extraction Kit (Qiagen, Hilden, Germany). DNA amplicons were sequenced on a MiSeq Illumina platform (Illumina, San Diego, CA, USA). Sequence outputs were analyzed using the Quantitative Insights into Microbial Ecology (QIIME) program, version 1.9.1 [27], using QIIME default parameters except for split library demultiplexing (phred quality threshold of 20 and better). The 16S pair-end reads were assembled using the script multiple_join_paired_ends.py, which joins forward and reverse demultiplexed reads. The output file was processed for quality filtering by split_libraries_fastq.py. High-quality sequences were grouped into Operational Taxonomic Units (OTUs) with a sequence identity threshold of 97%, and taxonomy was assigned by interrogating the high-quality sequences with the Greengenes database (13_8). Beta-diversity was evaluated by calculating weighted and unweighted Unifrac distances [28]. To study alpha-diversity, Shannon and Simpson diversity indices were calculated. Linear discriminant analysis (LDA) coupled with effect size (LEfSe) was performed to identify bacterial taxa differentially represented between groups [29]

### 2.7. Quantitative PCR Analysis

Quantitative PCR (qPCR) analysis was carried out to establish the relative abundance of *Bifidobacterium longum* [15], *Roseburia hominis* [15], *Faecalibacterium prautznii* [30], and *Bilophila wadsworthia* [31], on a CFX Connect™ Real-Time PCR Detection System (BioRad, Barcelona, Spain) using SYBR Green I chemistry (BioRad, Barcelona, Spain) in 20 μL reactions containing 10 ng of DNA template and 200 nM of primers. Cycling parameters were 95 °C for 10 min, followed by 40 cycles at 95 °C for 15 s, 1 min at the established annealing temperature and 72 °C for 45 s. Subsequently, melting curve analysis was performed, in which fluorescence was measured as the temperature increased from 50 °C to 95 °C. For bacterial quantification, standard curves were generated using serial dilutions of DNA corresponding to a known number of bacteria for each group or bacterial species cultivated under anaerobic conditions [32]. The calculation of the percentage for each bacterial species or group was performed considering the quantity of bacteria obtained per gram of stool obtained with a universal primer pair as 100% [33].

### 2.8. Fecal Water, pH, and Ammonia Content

To determine the fecal water content, a 500 mg fecal sample was weighed before and after lyophilization in a freeze-drier (Christ Alpha 1 ± 2 LD, Martin Christ Gefriertrocknungsanlagen GmbH, Osterode am Harz, Germany). Water content was expressed as the percentage of weight loss of stool samples. Fecal sample pH was determined with a basic 20+ Crison pH meter (Hach Lange, Barcelona, Spain) according to the method described by Dai & Karring [34]. Ammonia content was determined using a high-performance ammonia selective ion electrode (Orion™, Thermofisher Scientific, Waltham, MA, USA). To this end, 100 mg of feces was dissolved in 5 mL of MilliQ water and vortexed and sonicated (Ultrasons, Selecta, Barcelona, Spain) for 10 min. Samples were alkalized by the addition of 100 µL 1 M NaOH and immediately measured. A standard curve was obtained using serial dilutions of 0.1 M ammonium chloride according to the manufacturer’s instructions.

### 2.9. Short-Chain Fatty Acids

Fecal SCFAs were extracted according to the protocol described by García-Villalba et al. [35]. For sample analyses, 1 μL of the obtained supernatants was injected into an Agilent GC System 7820A chromatograph equipped with a DBWax 121-7037LT column and an Agilent Series MSD 5975 detector (Agilent Technologies, Inc. Santa Clara, CA, USA). Data acquisition was performed by selective ion monitoring. The target ions and qualifying ions are shown in Table 2. SCFAs were quantified using the peak area of their target ions against an eight-point external calibration curve (0.02 to 5.00 ppm) of reference standards (Sigma-Aldrich, St. Louis, MO, USA). 4-Methylvaleric acid was used as an internal standard.

### 2.10. Urine and Plasma Thiobarbituric Acid-Reacting Substances

Lipid peroxidation levels were measured in plasma and urine by the thiobarbituric-reactive substances (TBARS) method using the Malondialdehyde Assay Kit according to the manufacturer’s instructions (Northwest Life Science Specialties, Vancouver, WA, USA). Briefly, the samples or standards (250 µL) were incubated at 60 °C in a dry-block with 500 µL of thiobarbituric (TBA) reagent containing 2-thiobarbituric acid and butylhydroxytoluene for 60 min. Then, samples were centrifuged for 3 min at 10000× *g* and 200 µL of sample/standard were transferred to 96-well black plates. Fluorescence intensity (excitation 485 nm, emission 530 nm) was measured in a FP-8300 spectrofluorimeter (Jasco, Tokyo, Japan). To normalize TBARS levels in urine, creatinine was determined by the Jaffé method according to the protocol described by Junge et al. [36]. Data are expressed in terms of malondialdehyde equivalents, using malondialdehyde obtained from the hydrolysis of tetraethoxypropane (Sigma-Aldrich, St. Louis, MO, USA) as a standard.

### 2.11. Statistical Analysis

Statistical analysis was carried out using QIIME version 1.9.1, SPSS software 21.0 (SPSS, Chicago, IL, USA) and the R statistical package 3.3.1. Variable normal distribution was assessed using the Shapiro–Wilk test; when normal distribution was not assumed, non-parametric tests were performed. Intergroup comparisons of variables were performed with a *t*-test or Mann–Whitney test, and within-group comparisons were conducted with a paired *t*-test or Wilcoxon signed-rank test. Principal Component Analysis (PCA) of community structure (β-diversity) using the unweighted and weighted distance metric was generated by QIIME, visualized by EMPeror [27], and analyzed by permutational multivariate analysis of variance (PERMANOVA) using the script compare_categories.py. Statistical analysis of taxonomical data was performed only when the phylum, family, or genus was detected in 100% of the samples. LDA coupled with effect size (LEfSe) was performed to identify bacterial taxa differentially represented within and between groups at genus or higher taxonomy levels [29]. The significance was set initially at *p* < 0.05 and data were corrected with the Benjamini–Hochberg false discovery rate (FDR) *q*-value < 0.1 when necessary.

## 3. Results

### 3.1. Subjects and Body Composition

From the 24 volunteers recruited, 18 completed the study: 10 in the PRO group and eight in the CHO group. Six individuals dropped out for personal reasons (antibiotic intake, no-shows, time constraints, and vacations). There was no difference in age, BMI, and body composition parameters at baseline between the groups (Table 3).

### 3.2. Dietary Intake

Excluding nutrients provided by supplements, no changes in dietary habits were detected in volunteers’ diets, nor were differences detected in the energy intake, protein, carbohydrate, or total fat intake between *t* = 0 week and *t* = 10 weeks within each group or between groups (Table 4). When the protein and carbohydrate supplements were taken in consideration, an increase of 16.65 ± 3.16% of the baseline total protein intake in the PRO group and an increase of 7.53 ± 2.03% of the carbohydrate intake in the CHO group were found (Table 5). While this increment did not result in a significant increase in the total carbohydrate intake in the CHO group, supplementation with beef and whey protein blend caused a significant (*p* = 0.018) increase in the protein intake (total grams) in the PRO group, which was more marked when the data were expressed as kcal provided by protein (*p* = 0.003), grams per kg of body weight, or kcal provided per kg of body weight (*p* = 0.008) (Table 5). 

### 3.3. Fecal Water Content, pH, and Ammonia

Supplement consumption did not affect fecal water content in the CHO group (*t*0 = 42.74 ± 16.12%; *t*10w = 41.51 ± 13.93%; *p* = 0.842) or in the PRO group (*t*0 = 46.43 ± 9.72%; *t*10w = 43.05 ± 11.08%; *p* = 0.311). Fecal pH of the CHO group was 7.45 ± 0.47 at baseline and 7.70 ± 0.35 post-intervention, whereas in the PRO group it was 7.8 ± 0.43 before protein supplementation and 7.91 ± 0.57 at the end of the intervention period. All values were maintained at neutral pH ranges, and no significant changes were detected in the CHO (*p* = 0.123) or PRO (*p* = 0.541) groups after supplementation. Similarly, there were no significant changes in fecal concentrations of ammonia in the CHO (*t*0 = 15.06 ± 4.65 mmol/kg; *t*10w = 15.56 ± 2.91 mmol/kg; *p* = 0.700) or PRO (*t*0 = 13.64 ± 3.83 mmol/kg; *t*10w = 15.91 ± 3.83 mmol/kg; *p* = 0.224) groups. No significant differences were detected in fecal water (t = 0, *p* = 0.557; *t* = 10 weeks, *p* = 0.799) pH (*t* = 0, *p* = 0.126; *t* = 10 weeks, *p* = 0.378) or ammonia (*t* = 0, *p* = 0.523; *t* = 10 weeks, *p* = 0.832) when CHO and PRO groups were compared.

### 3.4. Short-Chain Fatty Acids

There were no differences within or between groups for fecal levels of SCFAs (acetic acid, propionic acid, butyric acid, isobutyric acid, valeric acid, and isovaleric acid), before or after the intervention (Table 6).

### 3.5. Plasma and Urine TBARS

No change in plasma or urine TBARS levels was detected after supplementation in the CHO or PRO groups: urine TBARS levels were *t*0 = 0.66 ± 0.42 µmol/g creatinine, *t*10w = 0.74 ± 0.49 µmol/g creatinine for the CHO group, and *t*0 = 0.73 ± 0.52 µmol/g creatinine, *t*10w = 0.83 ± 0.54 µmol/g creatinine for the PRO group. Similar plasma malondialdehyde levels were found before and after intervention in CHO and PRO groups (CHO: *t*0 = 1.56 ± 0.32 µM, *t*10w = 1.49 ± 0.27 µM; PRO: *t*0 = 1.51 ± 0.29 µM, *t*10w = 1.67 ± 0.34 µM).

### 3.6. Fecal Microbiota

**Figure 1:**
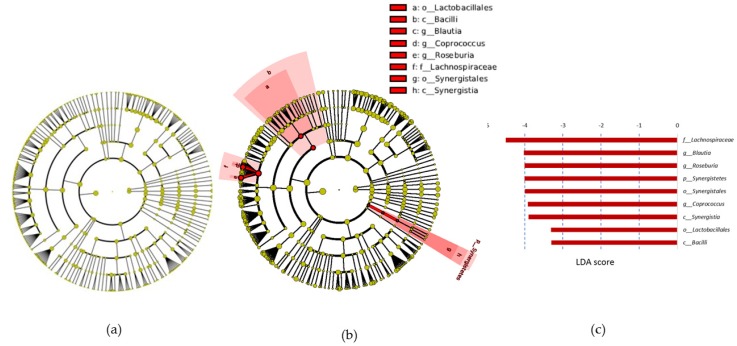
Cladograms and LDA (Linear Discriminant Analysis) score generated by LEfSe (Linear Discriminant Analysis Effect Size) indicating differences in bacterial taxa after CHO (maltodextrin group) and PRO (protein group) supplementation. The central point represents the root of the tree (bacteria), and each ring represents the next lower taxonomic level (phylum through genus). The diameter of each circle represents the relative abundance of the taxon. Red indicates those bacterial taxa that were significantly less abundant after 10 weeks of PRO supplementation. (**a**) Differences in bacterial taxa between (t0) and (t10w) in CHO group; (**b**) differences in bacterial taxa between (t0) and (t10w) in PRO group; (**c**) PRO LDA score; only the taxa meeting a significant LDA threshold value of >2 are shown. The PRO-reduced taxa are indicated with a negative LDA score.

The average number of reads per sample was 54,130. Rarefaction curves based on observed species, Chao1 diversity estimator index, and phylogenetic distance whole tree measures were virtually saturated, indicating sufficient sequencing depth (data not shown). When community complexities were studied, no significant differences were found between groups at baseline or after treatments. No significant differences were found in α-diversity parameters: Chao 1, equitability, phylogenetic diversity tree, number of observed species, Shannon and Simpson indices, or in β-diversity: PCA, based on unweighted Unifrac distance metrics (data not shown). Twelve different phyla were detected in the fecal microbiota, but only four phyla were present in all participants (*Firmicutes*, *Bacteroidetes*, *Actinobacteria*, and *Proteobacteria*). Within-group analysis with LEfSe revealed no significant changes in the CHO group (Figure 1a), whereas in the PRO group there was a significant decrease in the relative abundance of *Synergistetes* phylum, *Synergistales* order, and *Synergistia* class (Figure 1b). The most conspicuous decrease occurred in the *Lachnospiraceae* family (LDA score −4.5), followed by the *Roseburia*, *Blautia*, and *Coprococcus* genera (Figure 1b,c).

**Figure 2:**
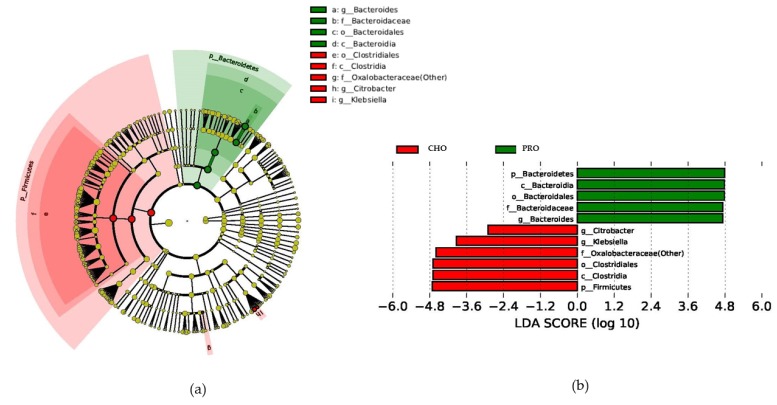
Cladogram and LDA score generated by LEfSe indicating differences in bacterial taxa between CHO and PRO groups after 10 weeks of supplementation. (**a**) Cladogram representing taxa enriched in PRO (green) and CHO (red) groups; (**b**) CHO and PRO LDA score; only the taxa meeting a significant LDA threshold value of >2 are shown. The CHO-enriched taxa are indicated in red (negative score), whereas the PRO-enriched taxa are indicated in green (positive LDA score).

No significant changes were found at the beginning of the study (*t* = 0) between PRO and CHO groups. After the 10-week intervention, CHO versus PRO group statistical analyses showed a total of 11 differentially abundant bacterial taxa. Of those, six were less abundant and five taxa were enriched in the PRO group as compared with the CHO group. At the phylum level, compared with the CHO group, athletes in the PRO group presented a higher abundance of the *Bacteroidetes* phylum and a lower abundance of the *Firmicutes* phylum (Figure 2a,b). At the genus level, a higher percentage of the *Bacteroides* genus and a lower presence of *Citrobacter and Klebsiella* genera were detected in the PRO group with respect to the CHO group (Figure 2a,b).

### 3.7. Quantification of Specific Bacterial Species by qPCR

Three health-related bacterial species (*B. longum*, *F. prautznii*, and *R. hominis*) and the meat consumption-related *B. wadsworthia* bacterium were quantified by qPCR. No changes in the relative abundance of any of the bacterial species determined were detected in the CHO group after the intervention. By contrast, the abundance of *B. longum* was 3.8-fold lower in the PRO group after the intervention (*t*0 = 0.057 ± 0.16%; *t*10w = 0.015 ± 0.05%; *p* = 0.021). Protein supplementation did not affect the relative abundance of *B. wadsworthia*, *F. prautznii*, or *R. hominis* bacteria.

## 4. Discussion

Gut microbiota can affect various health parameters in athletes, including immune function, weight management, psychological health, musculoskeletal conditions, asthma, and allergies [37], among others, and could be an important determinant for health and exercise performance. To our knowledge, this is the first study to examine the effect of a protein supplement on the gut microbiota of athletes. The main finding is that 10 weeks consumption of a protein supplement, which results in a small but significant increase in the daily protein intake of athletes, induces significant changes in the composition of the gut microbiota without affecting metabolite concentrations (SCFAs, ammonia) or other environmental parameters (pH, water content).

Professional and amateur athletes alike take dietary supplements to help improve their performance. Protein supplementation is popular among athletes because it increases muscle mass and strength gains during endurance exercise [38]. Moreover, protein overfeeding in resistance-trained athletes has been associated with lower malondialdehyde levels, a marker of oxidative stress [39]. The small increment in protein supplementation used in this study was not sufficient to reduce lipid oxidative stress, but nevertheless induced changes in microbiota. Elevations in protein intake can increase the amounts reaching the colon, where they are metabolized by colonic microbiota, leading to changes in microbiota populations and in microbial metabolites [40]. In a previous study, dietary protein intake and exercise was associated with a higher diversity of gut microbiota in professional rugby players [16], whereas in our study, no changes in microbiota diversity were detected after the intervention. One reason for this difference might be that the increase in the percentage of protein intake was insufficient to produce changes in microbial diversity. In line with our results, no significant changes were found in microbiota diversity after soy or casein protein supplementation (15% increase) in overweight humans [41]. By contrast, in an animal study, a considerable increase in protein intake (from 14% to 54%) was found to decrease colon microbiota diversity [42]. A possible explanation for this difference may be that the increase in protein intake in our study was insufficient to produce changes in microbial diversity (as it was increased from 20 to 22.5%). Another factor that influences the microbiota is the source of the protein: proteins from vegetable origin have a more marked effect on microbiota than animal proteins do [43]. In our study, the proteins were of animal origin—a blend of isolated whey proteins and hydrolyzed beef protein. Also, the fact that the protein was hydrolyzed may also influence the results since protein digestibility affects the amount of protein that reaches the colon [44].

Lefse analysis revealed an increase in the relative abundance of the *Bacteroidetes* phylum in the PRO group, which is consistent with the fact that species belonging to this phylum have proteolytic activity [45,46], and some *Bacteroides spp*. can use the urea from protein catabolism as a source of nitrogen [47]. It is thus possible that the elevated protein contribution through the supplement would increase substrate availability for these bacteria, supporting their growth over other bacteria of the phylum *Firmicutes*. Indeed, an increment in *Bacteroidetes* abundance has been previously associated with protein consumption [7], and could represent a benefit to human health because a shift in the *Bacteroidetes*/*Firmicutes* ratio with a reduced *Bacteroidetes* proportion has been associated with higher energy harvest, obesity, and chronic disorders [48]. By contrast, in the aforementioned observational study in rugby players, whose protein consumption accounted for 22% of their total energy intake, the authors found a decrease in the *Bacteroidetes* phylum when compared with that of the microbiota of healthy controls (whose protein consumption accounted for 15% of their total energy intake) [16]. Nevertheless, the decrease in *Bacteroidetes* abundance could have been due to the differences in physical exercise between healthy controls and rugby players [16].

At the genus level, we observed increases in the *Bacteroides* genus in the PRO group. This genus has been previously associated with proteolytic activity in the large intestine [49,50], which supports the hypothesis that more protein and subsequently more substrates for these group of bacteria were available in the PRO group. A reduction in lactobacilli, bifidobacteria, and butyrate-producing bacteria has been previously observed in diets with increased protein [40]. Accordingly, in our study, within-group analysis revealed a decrease in the SCFA producers *Coprococcus*, *Roseburia*, and *Blautia* genera and in *B. longum*. Although *Roseburia*, *Coproccus*, and *Blautia* genera are SCFA producers [51], the decline in their abundance was not reflected in changes to fecal SCFA levels. This could be because the observed changes in the microbiota are not sufficient to alter SCFA production, as the abundance of other SCFA producers such as *F. prautznii* was maintained at the two time-points. Alternatively, it is possible that SCFA production relies on other factors that were not controlled, such as intestinal transit time and time since last meal [52]. The intake of high-protein, low-carbohydrate diets has been linked to a decrease in SCFAs, an increase in pH, ammonia, and branched SCFAs, and deleterious effects on colon health [53,54]. In our study, the increase in protein consumption failed to induce changes in pH, ammonia, or branched fatty acids, which is in agreement with the results of Brinkworth et al., who did not find any significant change in fecal ammonia or pH after an eight-week protein diet intervention in humans [55]. Moreover, beneficial or deleterious effects of bacterial metabolites clearly depend on the concentration at which they are produced and their systemic absorption, and the concentration at which they have positive or negative effects on health, which is not well established [56].

It is of note that the observed decrease in bacterial populations in the PRO group were those of health-related taxa such as *Blautia*, *Roseburia*, and *Bifidobacterium longum* [57,58]. A decrease in *Roseburia* genus has been previously associated with animal-based diets and high-protein diets [53,59,60], whereas little is known about the effects of diet protein in the *Blautia* genus or *Bifidobacterium longum*. Nevertheless, it is worth mentioning that *Roseburia* and *Blautia* genera are implicated in SCFA production, whose health benefits have been substantially demonstrated [54], and that some strains of *Bifidobacterium longum* are used as probiotics for their purported health benefits [61]. Balancing the protein/carbohydrate ratio with prebiotics when protein intake is elevated [62], or accompanying the intake of protein supplements with probiotics, could be future strategies to mitigate the observed gut microbiota dysbiosis. Indeed, it has been recently described that the effect of protein supplements in recovery and alleviation of muscle soreness can be potentiated by taking probiotics [63].

## 5. Conclusions 

In conclusion, a slight increase in protein intake as a consequence of 20 g of protein supplementation (10 g of whey isolate + 10 g of beef hydrolysate) in athletes decreases health-promoting bacteria in microbiota without affecting SCFAs, ammonia, or fecal pH. Effects of protein supplementation for microbiota in athletes should be taken into consideration since nutritional recommendations regarding protein consumption in this population are higher than for the general population. The long-term consequences of the decrease in these bacterial taxa (*Blautia*, *Roseburia*, and *Bifidobacterium longum*) for gut health are unknown. Given the popularity of sport supplements, further research is warranted to determine how different doses, sources of protein, and dietary ratios of protein/carbohydrate affect athletes’ microbiota, and their potential health effects.

## Figures and Tables

**Table 1:** Nutrient composition of supplements and orange drink.

Nutritional Components	Maltodextrin	Protein	Orange Drink
Energy (kcal)	91.6	89.9	113.2
Carbohydrates (g)	22.9	0.4	27.9
Protein (g)	0	19.8	0.4
Fat (g)	0	1.0	0
Fiber (g)	0	0	0

**Table 2:** Mass of target ion and qualifier ions used to identify short-chain fatty acids (SCFAs) and calibration curve data for the standards.

SCFA	Target Ion (m/z)	Qualifier Ions (m/z)	Calibration Curve	r^2^
Acetic acid	43	45; 60	y = 9.558 × 10^−4^ + 1.693 × 10^−4^	0.999210
Propionic acid	74	73; 57	y = 5.977 × 10^−4^ + 3.041 × 10^−2^	0.999856
Butyric acid	73	60	y = 7.540 × 10^−4^ + 2.692 × 10^−3^	0.999797
Isobutyric acid	73	88	y = 8.346 × 10^−4^ + 3.447 × 10^−2^	0.999840
Valeric acid	73	60	y = 1.228 × 10^−5^ + 5.442 × 10^−2^	0.999841
Isovaleric acid	87	60	y = 5.519 × 10^−4^ + 2.783 × 10^−2^	0.999773
Methylvaleric acid	73	74; 83		

**Table 3:** Baseline characteristics of subjects.

Parameters	CHO *n* = 8	PRO *n* = 10	*p*
Age (years)	35.38 ± 9.00	34.90 ± 9.49	0.915
Body weight	70.07 ± 5.66	68.03 ± 4.91	0.424
BMI	23.37 ± 2.44	22.11 ± 1.03	0.205
BFP (%)	11.85 ± 4.31	10.41 ± 2.72	0.402
BFM (kg)	15.18 ± 5.19	13.06 ± 3.37	0.311
VAT (g)	364.20 ± 180.61	333.70 ± 134.47	0.717
AI (kg/m^2^)	5.41 ± 1.93	4.59 ± 1.01	0.304
MMI (kg/m^2^)	17.33 ± 1.22	17.07 ± 0.89	0.600
FFMI (kg/m^2^)	15.56 ± 1.42	15.19 ± 1.10	0.541

BMI: body mass index; BFP: body fat percentage; BFM: body fat mass; VAT: estimated visceral fat; AI: adiposity index; MMI: muscular mass index; FFMI: fat-free mass index. Values are means ± standard deviation. CHO: maltodextrin group; PRO: protein group.

**Table 4:** Nutrient intake per day without nutritional supplementation.

Diet Intake	CHO			PRO			CHO vs. PRO *t* = 0	CHO vs. PRO *t* = 10 Weeks
	*t* = 0	*t* = 10 Weeks	*p*	*t* = 0	*t* = 10 Weeks	*p*	*p*	*p*
Energy (kcal)	2735 ± 735	2576 ± 702	0.300	2617 ± 706	2693 ± 847	0.613	0.735	0.753
Carbohydrates (g)	282.58 ± 87.27	255.25 ± 68.78	0.155	286.73 ± 95.85	300.63 ± 98.06	0.306	0.926	0.319
Protein (g)	130.78 ± 31.03	128.76 ± 27.30	0.700	127.05 ± 24.61	128.76 ± 27.30	0.969	0.779	0.992
Fat (g)	116.01 ± 34.51	111.41 ± 37.05	0.682	103.23 ± 29.18	105.63 ± 37.74	0.821	0.407	0.749
Fiber (g)	21.26 ± 5.65	20.91 ± 4.57	0.576	20.91 ± 4.57	19.34 ± 3.46	0.496	0.607	0.419
Carbohydrates (%) of energy	41.37 ± 4.10	40.37 ± 4.80	0.582	43.80 ± 5.13	44.80 ± 5.76	0.653	0.294	0.102
Protein (%) of energy	19.62 ± 2.87	20.37 ± 1.99	0.365	20.00 ± 2.53	19.70 ± 2.31	0.591	0.773	0.319
Fat (%) of energy	38.87 ± 4.91	39.00 ± 4.86	0.949	36.00 ± 4.18	35.60 ± 4.94	0.844	0.199	0.164
Carbohydrates (g/kg bw)	4.03 ± 1.16	3.68 ± 0.95	0.201	4.20 ± 1.30	4.45 ± 1.40	0.290	0.783	0.201
Protein (g/kg bw)	1.86 ± 0.39	1.84 ± 0.34	0.809	1.87 ± 0.38	1.90 ± 0.39	0.790	0.960	0.766
Fat (g/kg bw)	1.66 ± 0.52	1.59 ± 0.49	0.656	1.52 ± 0.44	1.58 ± 0.51	0.715	0.550	0.948
Fiber (g/kg bw)	0.31 ± 0.09	0.30 ± 0.08	0.757	0.29 ± 0.08	0.29 ± 0.06	0.760	0.797	0.719
Carbohydrates (kcal/kg bw)	16.14 ± 4.64	14.73 ± 3.82	0.201	16.80 ± 5.20	17.83 ± 5.60	0.290	0.783	0.201
Protein (kcal/kg bw)	7.46 ± 1.57	7.39 ± 1.37	0.809	7.49 ± 1.50	7.60 ± 1.56	0.790	0.960	0.766
Fat (kcal/kg bw)	14.96 ± 4.76	14.34 ± 4.39	0.656	13.70 ± 3.96	14.20 ± 4.63	0.715	0.550	0.948

*t* = 0: initial time; *t* = 10 weeks: after 10 weeks of supplement consumption; bw: body weight. CHO: maltodextrin group; PRO: protein group. Values are means ± standard deviation.

**Table 5:** Nutrient intake per day after nutritional supplementation.

Diet Intake	CHO			PRO			CHO vs. PRO *t* = 0	CHO vs. PRO *t* = 10 Weeks
	*t* = 0	*t* = 10 Weeks	*p*	*t* = 0	*t* = 10 Weeks	*p*	*p*	*p*
Energy (kcal)	2735 ± 735	2656 ± 702	0.849	2617 ± 706	2783 ± 847	0.583	0.735	0.525
Carbohydrates (g)	282.58 ± 87.27	275.25 ± 68.78	0.681	286.73 ± 95.85	300.63 ± 98.06	0.306	0.926	0.573
Protein (g)	130.78 ± 31.03	128.76 ± 27.30	0.700	127.05 ± 24.61	148.76 ± 27.30	**0.018**	0.779	0.202
Fat (g)	116.01 ± 34.51	111.41 ± 37.05	0.682	103.23 ± 29.18	106.63 ± 37.74	0.748	0.407	0.791
Fiber (g)	21.26 ± 5.65	20.91 ± 4.57	0.576	20.91 ± 4.57	19.34 ± 3.46	0.496	0.607	0.419
Carbohydrates (%) of energy	41.37 ± 4.10	41.82 ± 4.92	0.812	43.80 ± 5.13	42.76 ± 5.42	0.607	0.294	0.709
Protein (%) of energy	19.62 ± 2.87	20.33 ± 2.14	0.414	20.00 ± 2.53	22.57 ± 3.09	**0.003**	0.773	0.102
Fat (%) of energy	38.87 ± 4.91	39.00 ± 4.97	0.949	36.00 ± 4.18	36.60 ± 4.95	0.749	0.199	0.319
Carbohydrates (g/kg bw)	4.03 ± 1.16	3.97 ± 0.96	0.811	4.20 ± 1.30	4.45 ± 1.40	0.642	0.783	0.416
Protein (g/kg bw)	1.86 ± 0.39	1.84 ± 0.34	0.809	1.87 ± 0.38	2.20 ± 0.39	**0.008**	0.960	0.059
Fat (g/kg bw)	1.66 ± 0.52	1.59 ± 0.49	0.656	1.52 ± 0.44	1.59 ± 0.51	0.642	0.550	0.998
Fiber (g/kg bw)	0.31 ± 0.09	0.30 ±0.08	0.757	0.29 ± 0.08	0.29 ± 0.06	0.760	0.797	0.719
Carbohydrates (kcal/kg bw)	16.14 ± 4.64	14.73 ± 3.82	0.811	16.80 ± 5.20	17.83 ± 5.60	0.642	0.783	0.416
Protein (kcal/kg bw)	7.46 ± 1.57	7.39 ± 1.37	0.809	7.49 ± 1.50	8.81 ± 1.54	**0.008**	0.960	0.059
Fat (kcal/kg bw)	14.96 ± 4.76	14.34 ± 4.39	0.656	13.70 ± 3.96	14.33 ± 4.62	0.642	0.550	0.998

*t* = 0: initial time; *t* = 10 weeks: after 10 weeks of supplement consumption; bw: body weight. CHO: maltodextrin group; PRO: protein group. Values are means ± standard deviation.

**Table 6:** Fecal short-chain fatty acids.

SCFA (µg/g)	CHO			PRO		
	*t* = 0	*t* = 10 Weeks	*p*	*t* = 0	*t* = 10 Weeks	*p*
Acetic acid	1425.30 ± 51.89	1532.25 ± 390.71	0.674	1493.69 ± 531.10	1379.55 ± 431.30	0.721
Propionic acid	894.11 ± 338.79	791.95 ± 420.49	0.401	949.78 ± 521.16	776.71 ± 349.31	0.575
Butyric acid	1169.94 ± 532.48	1084.52 ± 650.59	0.484	1240.91 ± 888.33	957.04 ± 474.89	0.241
Isobutyric acid	119.31 ± 48.54	90.85 ± 69.04	0.779	119.88 ± 47.11	111.87 ± 61.57	0.508
Valeric acid	210.43 ± 111.84	158.81 ± 96.25	0.484	171.12 ± 79.78	158.00 ± 85.92	0.285
Isovaleric acid	213.10 ± 94.33	162.51 ± 145.33	0.674	222.44 ± 99.41	212.55 ± 132.22	0.203

*t* = 0: initial time; *t* = 10 weeks: after 10 weeks of supplement consumption. Values are means ± standard deviation.
